
JOURNAL INFORMATION
==============================
NLM Title Abbreviation: ScientificWorldJournal
Journal ID: ScientificWorldJournal
NLM Journal ID: 101131163
Journal ID: TSWJ

The Scientific World Journal

ISSN: 2356-6140
EISSN: 1537-744X
Publisher: Wiley

ARTICLE INFORMATION
==============================
PMCID: PMC5936534
PMID: 15702223
DOI: 10.1100/tsw.2005.18
Article ID: 159481
Article version: 1
Subjects: Case Study

A Rare Case of an Extra-Adrenal Myelolipoma Arising in the Renal Sinus: A Case Report and Review of the Literature

Clark Peter E. 1 *
Farver Carol F. 2
Ulchaker James C. 3
Angermeier Kenneth 3
1 Department of Urology, Wake Forest University Health Sciences, Winston-Salem, NC 27157, USA
2 Department of Pathology, The Cleveland Clinic Foundation, Cleveland, OH, USA
3 Glickman Urological Institute, The Cleveland Clinic, Cleveland, OH, USA
*Peter E. Clark: peclark@wfubmc.edu
Publication date: 2005
Electronic publication date: 2005 Jan 28
Volume: 5
First page: 109
Last page: 117
Received 2004 Oct 20; Accepted 2005 Jan 17
Copyright: Copyright © 2005 Peter E. Clark et al.
Copyright year: 2005
License: This is an open access article distributed under the Creative Commons Attribution License, which permits unrestricted use, distribution, and reproduction in any medium, provided the original work is properly cited.
License URL: https://creativecommons.org/licenses/by/3.0/

Keywords: extra-adrenal myelolipoma, renal sinus

==============================
Case Study
*TheScientificWorldJOURNAL* (2005) 5, 109–117
ISSN 1537-744X; DOI 10.1100/tsw.2005.18

### A Rare Case of an Extra-Adrenal Myelolipoma Arising in the Renal Sinus: A Case Report and Review of the Literature

Peter E. Clark[1], Carol F. Farver[2], James C. Ulchaker[3], and Kenneth Angermeier[3]

[1]*Department of Urology, Wake Forest University Health Sciences, Winston-Salem, NC;* [2]*Department of Pathology, The Cleveland Clinic Foundation, Cleveland, OH;* [3]*Glickman Urological Institute, The Cleveland Clinic, Cleveland, OH*

**E-mail**: peclark@wfubmc.edu

*Received October 20, 2004; Accepted January 17, 2005; Published January 28, 2005*

**KEYWORDS**: extra-adrenal myelolipoma, renal sinus

**DOMAIN**: urology

### INTRODUCTION

Adrenal myelolipoma is a benign lesion composed of mature adipose tissue and myeloid elements that are generally asymptomatic and detected incidentally, either radiographically or at autopsy. It is a rare entity with an incidence on autopsy series of 0.08–0.2%[1]. Extra-adrenal myelolipomas are well described in the literature and usually found retroperitoneally in the presacral space[2]. They can also occur, however, in a variety of other locations including the stomach, mediastinum, spine, and liver[3,4,5,6,7,8,9,10,11,12,13]. A total of 16 cases of perirenal myelolipoma have been reported in the literature to date[2,14,15,16,17,18,19,20,21,22,23,24]. Of these, only one originated in the renal sinus[23]. We report only the second case, to our knowledge, of an extra-adrenal myelolipoma originating from the renal sinus and review the literature on this rare tumor.

### CASE REPORT

S.W. is a 59-year-old, Caucasian male who presented to the urology clinic with left flank and upper abdominal pain of 4 months duration associated with abdominal fullness, worsening wheezing, and dyspnea on exertion, poor appetite, and a 20-lb weight loss. He had no voiding symptoms and no hematuria.

The patient's past medical history was notable for sarcoidosis diagnosed 33 years previously (for which he was on long-term steroids), chronic obstructive pulmonary disease, type II diabetes mellitus, hypertension, gout, coronary artery disease, morbid obesity, and right phrenic nerve paresis. Physical exam demonstrated a morbidly obese male who had mild tenderness and fullness in the left upper abdomen.

*Corresponding author address: Department of Urology, Wake Forest University Health Sciences, Medical Center Boulevard, Winston-Salem, NC 27157; Tel: 336/716-5702/Fax: 336/716-5711
©2005 with author.

Laboratory studies showed normal electrolytes, a serum creatinine of 1.1 mg/dl, blood urea nitrogen of 19 mg/dl, and normal liver function tests. His complete blood count was normal, including a serum hemoglobin of 14.1 g/dl, hematocrit of 40.8 %, and a RBC volume of 4.47 m/µl.

A computerized tomography (CT) scan of the abdomen/pelvis demonstrated an 8.5-cm, left renal, hilar mass with fat attenuation felt to be consistent with an angiomyolipoma (see Fig. 1). Given the patient's overall medical condition, interventional radiologic embolization of the mass was attempted. An angiogram was obtained that demonstrated a large, relatively hypovascular mass projecting from the lower pole of the left kidney (see Fig. 2). What vascularity was present arose from small renal and cortical branches and remained at the periphery of the mass. No single arterial supply conducive to embolization could be identified. The procedure was terminated and it was elected to observe this lesion with serial imaging.

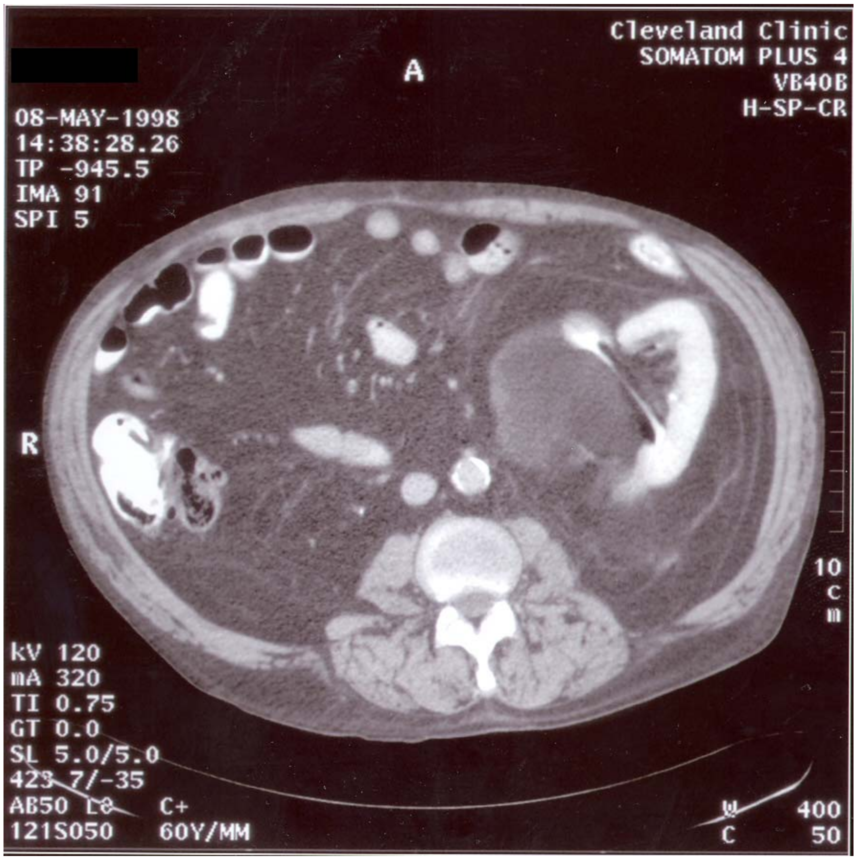

**FIGURE 1**. CT scan demonstrating an 8.5-cm, predominantly fat, attenuation mass arising from the left renal hilum with soft-tissue attenuation around its periphery. No adenopathy was identified.

Five months later, a repeat CT scan of the abdomen/pelvis demonstrated a left-sided, 9.0-cm mass with fat attenuation that it was felt may be retroperitoneal rather than hilar. A renal scan at that time demonstrated normal arterial flow with differential function of 55% on the right and 45% on the left. Given the increase in size and the concern for a possible liposarcoma, the patient was advised to undergo immediate exploration and surgical excision of the mass. The patient refused immediate surgery preferring to wait until the end of the following summer.

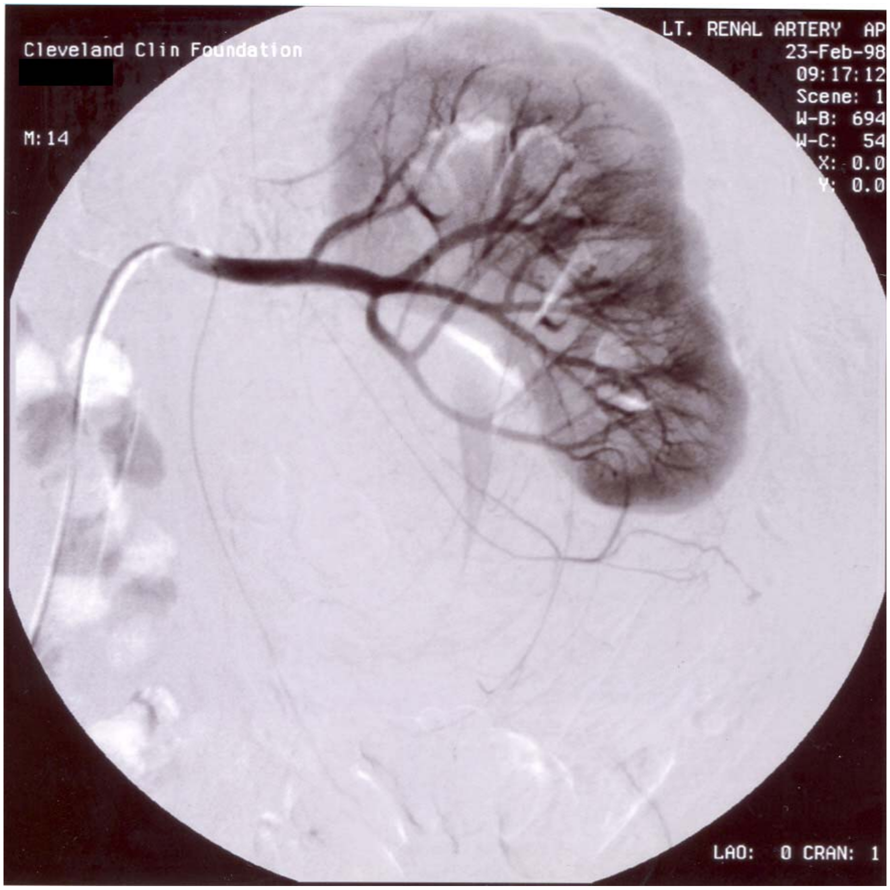

**FIGURE 2**. Renal angiogram demonstrating a large, hypovascular mass originating from the renal hilum.

Ten months after the initial presentation, the patient underwent magnetic resonance imaging (MRI) of the abdomen, which demonstrated a large, 9.2-cm, left-sided mass with the epicenter in the left renal sinus and apparently confined to gerota’s fascia (see Fig. 3). The T2 signal intensity was consistent with fat and the renal vein was patent. After medical optimization, the patient underwent surgical exploration via an anterior transabdominal approach. A large, left renal mass was noted and a left radical nephrectomy performed. Postoperatively, the patient did well and was discharged home in good condition on the eighth postoperative day.

Final pathology showed a 14.1- × 10.2- × 6.8-cm mass that replaced the lower pole of the left kidney and appeared to originate in the renal sinus. The mass was grossly circumscribed and sharply demarcated from the surrounding renal parenchyma, but nonencapsulated (see Fig. 4A). On cut section, the mass was yellow-red with an oily surface. There were areas of recent hemorrhage, however, no areas of necrosis were identified. Microscopic examination showed areas of mature adipose tissue and hematopoiesis consistent with myelolipoma originating in the left kidney (see Fig. 4B). Immunohistochemical staining of the lipomatous areas were negative for HMB-45 and smooth muscle actin. No adrenal rests were identified. The left adrenal gland was entirely normal. A final diagnosis of left, renal myelolipoma was returned.

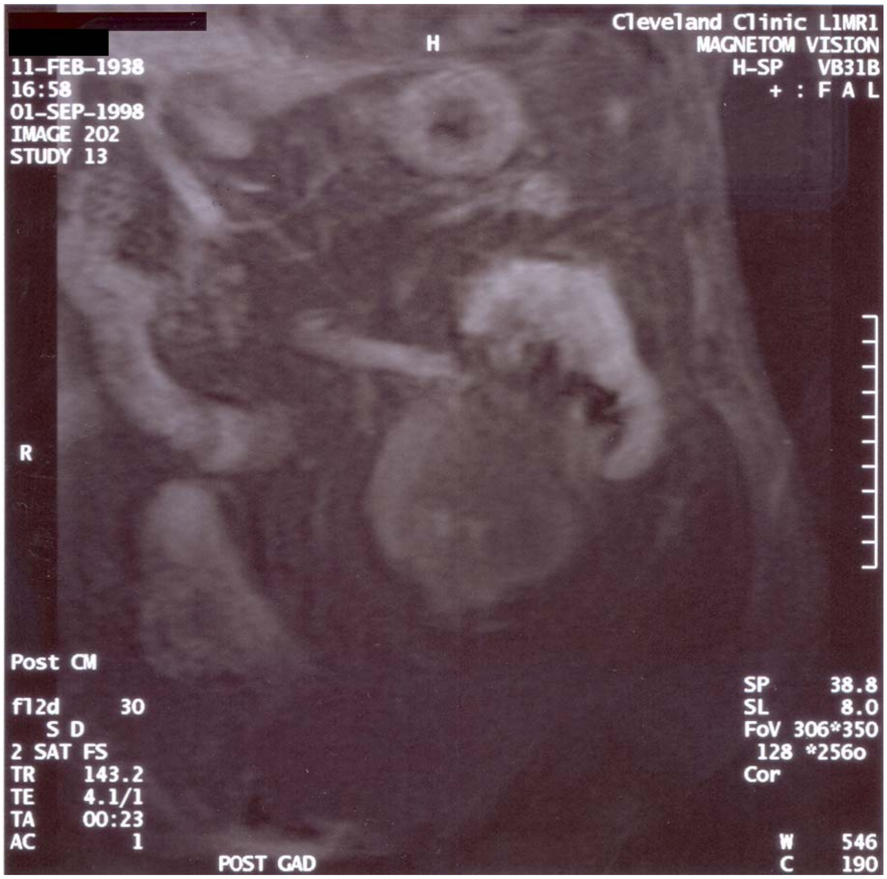

**FIGURE 3.** Abdominal MRI confirms adipose-containing mass emanating from the renal hilum with mild irregular peripheral enhancement.

### DISCUSSION

Myelolipomas are rare benign neoplasms that most commonly arise in the adrenal glands. They are composed of a variable mixture of mature adipose tissue and hematopoietic elements. Myelolipomas were first described by Gierke in 1905[25]. The term myelolipoma was coined by Oberling in 1929[26]. Since then, numerous reports of adrenal myelolipomas have appeared in the literature[20]. They are benign lesions with no reported cases of metastasis and are generally asymptomatic and detected incidentally either radiographically or at autopsy.

Myelolipomas outside of the adrenal gland (or extra-adrenal myelolipomas) are even more rare. These generally occur in the retroperitoneal space, and in particular in the presacral space, although cases of myelolipomas in the liver, stomach, and thoracic cavity have also been reported[3,4,5,6,7,8,9,10,11,12,13]. Perirenal, extra-adrenal myelolipomas have also been reported, with a total of 16 published cases in the English literature[2,14,15,16,17,18,19,20,21,22,23,24]. Only one of these tumors appears to originate in the renal sinus[23]. This case represents only the second reported case in the literature.

Myelolipomas in general are lucent on plain films and hyperechoic on ultrasound[27]. CT scan generally demonstrates a fatty tumor intermixed with areas of enhancing soft tissue density[20,28]. There may be a fibrous capsule and fibrous septations. These lesions are typically avascular by angiography, and MRI findings are consistent with a primarily adipose containing tumor[13,19,27,29]. The case presented here demonstrates several of these features.

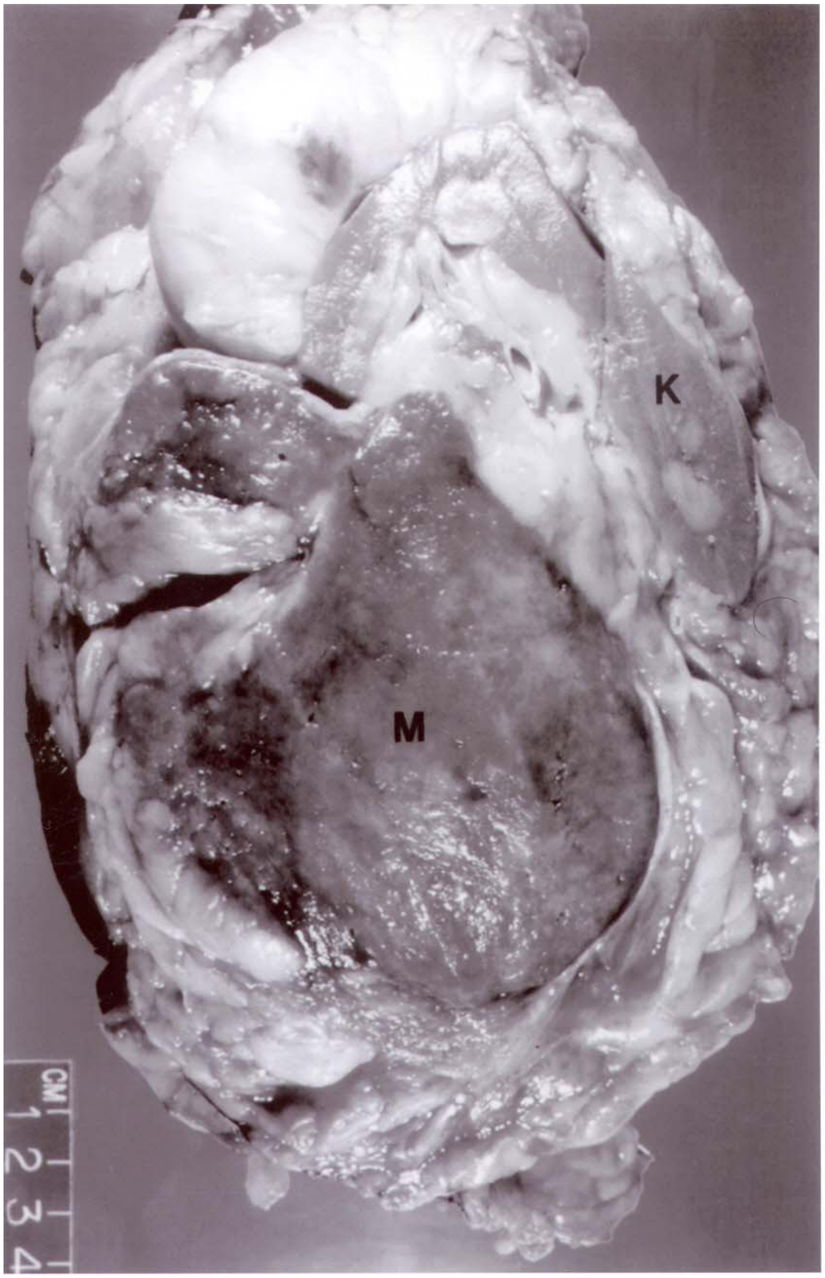

A

**FIGURE 4.** (A) Gross photograph of left radical nephrectomy specimen showing a 14.1-cm, tan/red mass (M) arising within the lower pole of the left kidney. Residual kidney (K) can be seen surrounded by perinephric adipose tissue. (B) Photomicrograph of myelolipoma showing characteristic adipose tissue with islands of bone marrow elements (hematoxylin and eosin: 400×).

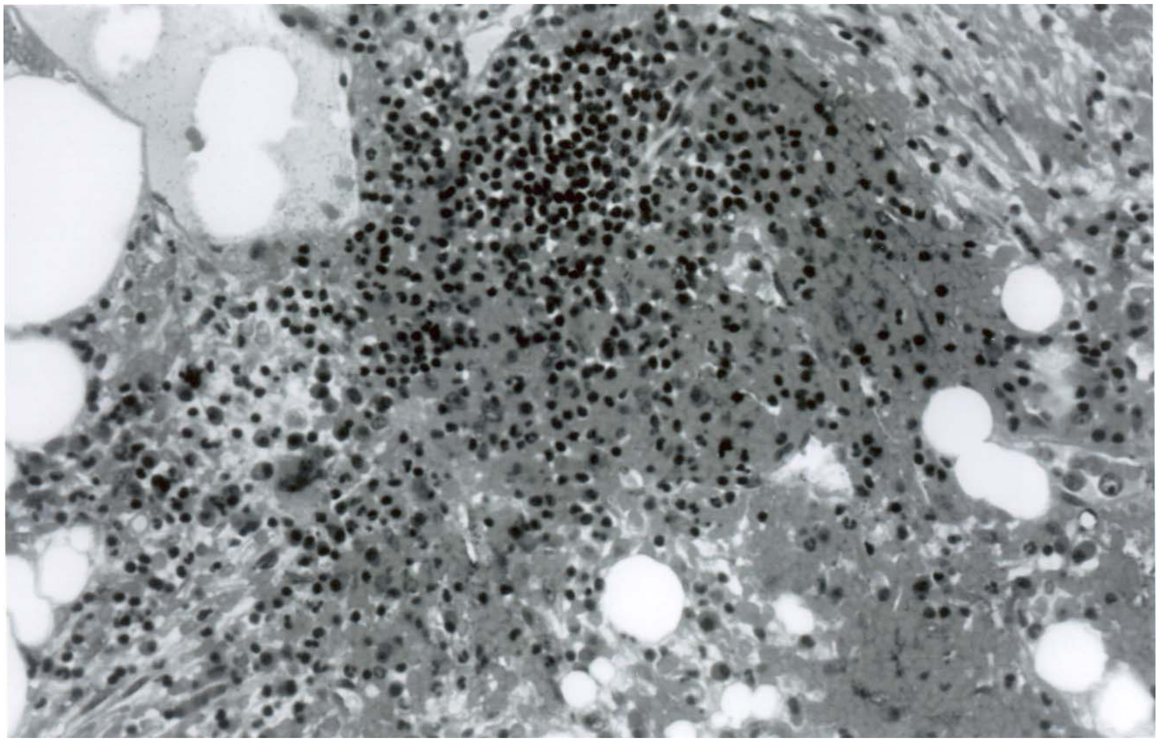

**FIGURE 4B**

Retroperitoneal extra-adrenal myelolipomas must be distinguished from other adipose-containing tumors of the retroperitoneum. These include both benign and malignant conditions such as angiomyolipoma, liposarcoma, lipoma, extramedullary hematopoiesis, and pelvic lipomatosis[14]. Although there have been cases of preoperative, fine-needle aspiration biopsy making the diagnosis, the majority of the time the diagnosis is made after surgical extirpation[8,30,31]. Fortunately, the various tumors in the differential diagnosis are generally easily distinguished pathologically. The distinction, however, between extra-adrenal myelolipoma and extramedullary hematopoiesis can be a difficult one. A report by Fowler et al. outlined the major differences between these two entities (see Table 1)[32]. The tumor in this report meets the criteria for extra-adrenal myelolipoma as outlined in Fowler's report. The patient was older (59 years old), with diabetes, coronary artery disease, morbid obesity, and on chronic steroids. The patient had no history of significant hematologic disorders including anemia. The tumor was solitary, well demarcated, with gross yellow areas on cut section, and a mixture of hematopoietic and mature adipose tissue, a significant proportion of which was fat. There were no adrenal rests, bony spicules, or continuity with the medullary bone cavity. Its origin from the renal sinus has only been reported once before, as all prior retroperitoneal cases have been perirenal or presacral. Nevertheless, the combination of features described above strongly supports the diagnosis of extra-adrenal myelolipoma originating from the renal sinus. The lesion did increase in size over time from 8.0–9.2 cm. over a 9-month time span, as has been reported previously for adrenal myelolipomas[33].

The etiology of myelolipomas remains obscure. Several theories exist including embolization of bone marrow elements, hyperplasia of embryonic bone marrow rests within the adrenal gland or elsewhere, metaplasia of adrenal cortical reticuloendothelial cells or undifferentiated stromal cells, or that they are hamartomas[2,15,20,30,32,33,34]. Extra-adrenal myelolipomas are generally thought to arise from rests of choristomatous intrabdominal hematopoietic stem cells that are stimulated to undergo hyperplasia and at times neoplasia[2,21,32,34]. Regardless of the cell of origin, the stimulus for hyperplasia or metaplasia is thought to be long-term exposure to stress and/or steroids[35,36]. This is supported by both

**TABLE 1**
**Differentiating Extra-Adrenal Myelolipoma vs. Extramedullary Hematopoietic Tumor**

| | Extra-Adrenal Myelolipoma | Extramedullary Hematopoietic Tumor |
|---|---|---|
| **Patient Characteristics** | | |
| Male:Female | 5:8 | 3:1 |
| Age range | 5th to 7th decade (none less than 40 years old) | 1st to 7th decade (10% in 1st decade) |
| Average age | 61 years | 43.7 years |
| Splenomegally/hepatomegally | Absent | Present |
| Associated hematologic disorder | Absent | Present (especially anemia) |
| Associated bone marrow hyperplasia | Absent | Present |
| **Tumor Characteristics** | | |
| Usual number | Solitary | Multiple |
| Usual location | Abdominal | Retropleurally lateral to vertebral column |
| Boney spicules | Absent | May be present |
| Connection to medullary bone cavity | Absent | May be present |
| Association with adrenal tissue | Absent | Absent |
| **Pathologic Characteristics** | | |
| Yellow fatty areas grossly on cut section | Common | Rare |
| Adipose:hematopoietic ratio | Adipose predominant in 70% | Adipose never predominant (two cases with no fat) |
| Myloid:erythroid ratio | Mainly lymphocyte aggregates | Erythroid hyperplasia |

Based on criteria outlined in Fowler et al.[32].

experimental and clinical data. Work by Selye and Stone showed that injection of crude anterior pituitary extracts rich in corticotropin into the adrenal glands of rats resulted in marked myelopoiesis[37]. Myelolipomas have been seen in patients with both Cushing's disease and congenital adrenal hyperplasia[38,39]. An increased incidence of myelolipomas has been noted at autopsy in patients with chronic debilitating diseases such as cancer or burn victims[1,40,41]. It has also been associated with other chronic debilitating diseases such as coronary artery disease, obesity, diabetes mellitus, and other endocrinopathies (including long-term exogenous steroid use), all of which were present in our patient[14,18,32].

In conclusion, we present the second reported case to our knowledge of an extra-adrenal myelolipoma that arose from the renal sinus. Typical radiographic and pathologic features of myelolipoma are presented. The patient's history of chronic debilitating disease and exogenous steroid use support the theory that exposure to long-term stress and/or steroids is important in the pathogenesis of this rare, benign neoplasm.

### REFERENCES

1. Olsson, C.A., Krane, R.J., Klugo, R.C., and Selikowitz, S.M. (1973) Adrenal myelolipoma. *Surgery* **73,** 665–670.
2. Brietta, L.K. and Watkins, D. (1994) Giant extra-adrenal myelolipoma. *Arch. Pathol. Lab. Med.* **118**, 188–190.
3. Foster, J.B. (1958) Primary thoracic myelolipoma: case report. *AMA Arch. Pathol.* **65**, 295–297.
4. Grosdidier, J., Boissel, P., Macinot, C., and Drouin, P. (1973) [Hepatic myelolipoma. Apropos of a case]. *Nouv. Presse Med.* **2**, 1777–1779.

5. Le Bodic, M.F., Mussini-Montpellier, J., Magois, J.Y., and Lepere, J. (1974) [Myelolipoma of the stomach]. *Arch. Anat. Pathol. (Paris)* **22,** 119–122.
6. Krag, D. and Reich, S.B. (1972) Heterotopic bone marrow (myelolipoma) of the mediastinum. *Chest* **61**, 514–515.
7. Kim, K., Koo, B.C., Davis, J.T., and Franco-Saenz, R. (1984) Primary myelolipoma of mediastinum. *J. Comput. Tomogr.* **8**, 119–123.
8. Spanta, R., Saleh, H.A., and Khatib, G. (1999) Fine needle aspiration diagnosis of extraadrenal myelolipoma presenting as a pleural mass. A case report. *Acta Cytol.* **43**, 295–298.
9. Sabate, C.J. and Shahian, D.M. (2002) Pulmonary myelolipoma. *Ann. Thorac. Surg.* **74,** 573–575.
10. Minamiya, Y., Abo, S., Kitamura, M., and Izumi, K. (1997) Mediastinal extraadrenal myelolipoma: report of a case. *Surg. Today* **27**, 971–972.
11. Kawanami, S., Watanabe, H., Aoki, T., Nakata, H., Hayashi, T., Kido, M., Tsukada, J., and Eto, S. (2000) Mediastinal myelolipoma: CT and MRI appearances. *Eur. Radiol.* **10**, 691–693.
12. Omdal, D.G., Baird, D.E., Burton, B.S., Goodhue, W.W., Jr., and Giddens, E.M. (1997) Myelolipoma of the thoracic spine. *AJNR Am. J. Neuroradiol.* **18**, 977–979.
13. Savoye-Collet, C., Goria, O., Scotte, M., and Hemet, J. (2000) MR imaging of hepatic myelolipoma. *AJR Am. J. Roentgenol.* **174**, 574–575.
14. Prahlow, J.A., Loggie, B.W., Cappellari, J.O., Scharling, E.S., Teot, L.A., and Iskandar, S.S. (1995) Extra-adrenal myelolipoma: report of two cases. *South. Med. J.* **88**, 639–643.
15. Sneiders, A., Zhang, G., and Gordon, B.E. (1993) Extra-adrenal perirenal myelolipoma. *J. Urol.* **150**, 1496–1497.
16. Wagner, J.R., Kleiner, D.E., Walther, M.M., and Linehan, W.M. (1997) Perirenal myelolipoma. *Urology* **49**, 128–130.
17. Shapiro, J.L., Goldblum, J.R., Dobrow, D.A., and Ratliff, N.B. (1995) Giant bilateral extra-adrenal myelolipoma. *Arch. Pathol. Lab. Med.* **119**, 283–285.
18. d'Addessi, A., Racioppi, M., Fanasca, A., Zannoni, G.F., and Alcini, E. (1997) A rare case of perirenal myelolipoma. Diagnostic approach and therapeutic strategy. *Scand. J. Urol. Nephrol.* **31,** 579–581.
19. Kammen, B.F., Elder, D.E., Fraker, D.L., and Siegelman, E.S. (1998) Extraadrenal myelolipoma: MR imaging findings. *AJR Am. J. Roentgenol.* **171**, 721–723.
20. Kenney, P.J., Wagner, B.J., Rao, P., and Heffess, C.S. (1998) Myelolipoma: CT and pathologic features. *Radiology* **208**, 87–95.
21. Hunter, S.B., Schemankewitz, E.H., Patterson, C., and Varma, V.A. (1992) Extraadrenal myelolipoma. A report of two cases. *Am. J. Clin. Pathol.* **97**, 402–404.
22. Beraha, D., Block, N.L., and Politano, V.A. (1974) Myelolipoma of the kidney. *J. Urol.* **112**, 19–21.
23. Amin, M.B., Tickoo, S.K., and Schultz, D. (1999) Myelolipoma of the renal sinus. An unusual site for a rare extra-adrenal lesion. *Arch. Pathol. Lab. Med.* **123**, 631–634.
24. Rozsahegyi, J., Szabo, F., Gonda, G., Fulop, R., Forrai, G., and Gamal, E.M. (2001) Perirenal myelolipoma causing giant, infected hydronephrosis and renal pelvic stone. *J. Urol.* **165,** 1620–1621.
25. Gierke, E. (1905) Uber Knochenmarksgebe in der Nebennierc. *Bietr. Pathol. Anat.* **7**, 311–324.
26. Oberling, C. (1929) Les formations myelo-lipomateuses. *Bull. Assoc. Fr. Cancer* **18**.
27. Asch, M.R., Poon, P.Y., McCallum, R.W., and Bird, B.I. (1989) Myelolipoma: radiologic findings in seven patients. *Can. Assoc. Radiol. J.* **40,** 247–250.
28. Sutker, B., Balthazar, E.J., and Fazzini, E. (1985) Presacral myelolipoma: CT findings. *J. Comput. Assist. Tomogr.* **9**, 1128–1130.
29. Chan, Y.F., Yu, S.J., Chan, Y.T., and Yik, Y.H. (1988) Presacral myelolipoma: case report with computed tomographic and angiographic findings. *Aust. N. Z. J. Surg.* **58**, 432–434.
30. Bryan, J.A., Sykes, C.H., and Garvin, D.F. (1996) Fine needle aspiration diagnosis of a mesenteric myelolipoma: a case report. *Acta Cytol.* **40**, 592–594.
31. Saboorian, M.H., Timmerman, T.G., Ashfaq, R., and Maiese, R.L. (1999) Fine-needle aspiration of a presacral myelolipoma: a case presentation with flow cytometry and immunohistochemical studies. *Diagn. Cytopathol.* **20**, 47–51.
32. Fowler, M.R., Williams, R.B., Alba, J.M., and Byrd, C.R. (1982) Extra-adrenal myelolipomas compared with extramedullary hematopoietic tumors: a case of presacral myelolipoma. *Am. J. Surg. Pathol.* **6**, 363–374.
33. Meaglia, J.P. and Schmidt, J.D. (1992) Natural history of an adrenal myelolipoma. *J. Urol.* **147**, 1089–1090.
34. Cina, S.J., Gordon, B.M., and Curry, N.S. (1995) Ectopic adrenal myelolipoma presenting as a splenic mass. *Arch. Pathol. Lab. Med.* **119**, 561–563.
35. Chen, K.T., Felix, E.L., and Flam, M.S. (1982) Extraadrenal myelolipoma. *Am. J. Clin. Pathol.* **78,** 386–389.
36. Oliva, A., Duarte, B., Hammadeh, R., Ghosh, L., and Baker, R.J. (1988) Myelolipoma and endocrine dysfunction. *Surgery* **103**, 711–715.
37. Selye, H. and Stone, H. (1950) Hormonally induced transformation of adrenal into myeloid tissue. *Am. J. Surg. Pathol.* **26**, 211–233.
38. Bennett, B.D., McKenna, T.J., Hough, A.J., Dean, R., and Page, D.L. (1980) Adrenal myelolipoma associated with Cushing's disease. *Am. J. Clin. Pathol.* **73**, 443–447.

39. Boudreaux, D., Waisman, J., Skinner, D.G., and Low, R. (1979) Giant adrenal myelolipoma and testicular interstitial cell tumor in a man with congenital 21-hydroxylase deficiency. *Am. J. Surg. Pathol.* **3**, 109–123.
40. Delarue, J. and Monsaingeon, A. (1950) [Myeloid metaplasia in the adrenal cortex of burned subjects]. *C. R. Seances Soc. Biol. Fil*. **44**, 777–778.
41. Gee, W.F., Chikos, P.M., Greaves, J.P., Ikemoto, N., and Tremann, J.A. (1975) Adrenal myelolipoma. *Urology* **5**, 562–566.

---

**This article should be referenced as follows:**

Clark, P.E., Farver, C.F., Ulchaker, J.C., and Angermeier, K. (2005) A rare case of an extra-adrenal myelolipoma arising in the renal sinus: a case report and review of the literature. *TheScientificWorldJOURNAL* **5**, 109–117.

**Handling Editor:**

Anthony Atala, Principal Editor for *Urology* and *Tissue Engineering*— domains of *TheScientificWorldJOURNAL*.

---
